# Characteristics of eating behavior profiles among preschoolers with low-income backgrounds: a person-centered analysis

**DOI:** 10.1186/s12966-022-01323-y

**Published:** 2022-07-23

**Authors:** Jennifer Orlet Fisher, Sheryl O. Hughes, Alison L. Miller, Mildred A. Horodynski, Holly E. Brophy-Herb, Dawn A. Contreras, Niko Kaciroti, Karen E. Peterson, Katherine L. Rosenblum, Danielle Appugliese, Julie C. Lumeng

**Affiliations:** 1 Center for Obesity Research and Education, College of Public Health, Temple University, Philadelphia, PA USA; 2 USDA/ARS Children’s Nutrition Research Center, Baylor College of Medicine, Houston, TX USA; 3 Department of Health Behavior and Health Education, School of Public Health, University of Michigan, Ann Arbor, MI USA; 4 College of Nursing, Michigan State University, East Lansing, MI USA; 5 Department of Human Development and Family Studies, Michigan State University, East Lansing, MI USA; 6 Health and Nutrition Institute, Michigan State University Extension, East Lansing, MI USA; 7 Department of Pediatrics, University of Michigan Medical School, Ann Arbor, MI USA; 8 Department of Nutritional Sciences, School of Public Health, University of Michigan, Ann Arbor, MI USA; 9 Department of Psychiatry, University of Michigan Medical School, Ann Arbor, MI USA; 10 Appugliese Professional Advisors, LLC, North Easton, MA USA

**Keywords:** Children, Eating behavior, Food approach, Food avoidance, Temperament, Obesity

## Abstract

**Background:**

Individual differences in eating behaviors among young children are well-established, but the extent to which behaviors aggregate within individuals to form distinct eating behavior profiles remains unknown. Our objectives were to identify eating behavior profiles among preschool-aged children and evaluate associations with temperament and weight.

**Methods:**

A secondary, cross-sectional analysis of baseline data from 2 cohort studies was conducted involving 1004 children aged 3–4 years and their parents with low-income backgrounds. Children’s eating behaviors and temperament were assessed by parental report. Body mass index *z*-scores and weight status were calculated using measured heights and weights. Latent profile analysis (LPA) was used to generate profiles and bivariate analyses were used to evaluate associations with temperament and weight status.

**Results:**

LPA revealed the presence of 3 eating behavior profiles among children. Children with High Food Approach profiles (21.2%) had lower temperamental inhibitory control and the highest percent of children with obesity relative to the other profiles. Children with High Food Avoidant profiles (35.6%) had lower temperamental impulsivity and lower BMI *z*-scores relative to the other profiles, whereas children with Moderate Eating profiles (intermediary levels of all behaviors; 43.2%) had higher temperamental inhibitory control and lower anger/frustration, than other profiles.

**Conclusions:**

Young children’s eating behaviors appear to aggregate within individuals to form empirically distinct profiles reflecting food approach, food avoidance, and moderate approaches to eating that are differentiated by aspects of temperament and weight. Future work should seek to understand the extent to which health promotion and obesity prevention approaches should be tailored to take into account children’s fundamental dispositions towards eating.

## Background

Eating behaviors have an integral role in human growth and development as well as the prevention of life-long chronic diseases, including obesity [1, 2]. More than a dozen eating behaviors have been described in children and have been suggested to broadly describe appetite self-regulation [3] tendencies towards food approach (e.g., food responsiveness) and avoidance (e.g., food neophobia) [3, 4].

Eating behaviors thought to represent food approach include enjoyment of food (general interest in food [4–6]), food responsiveness (responsiveness to external food cues [4–6]), eating in the absence of hunger (consumption of palatable foods when satiated [7]), and relative reinforcing value of food (how hard an individual is willing to work to gain access to food [8]). Child body mass index (BMI) has been positively associated with multiple food approach behaviors across studies involving preschool-aged children, including parental reports of enjoyment of food [9–13], food responsiveness [9, 13–15], and desire to drink as well as observed eating in the absence of hunger [7, 16–19] and food reinforcement (an individual’s willingness to work to gain access to food when an alternative reinforcer is available [20–22]) in laboratory protocols [8, 23].

Alternatively, eating behaviors thought to represent food avoidance include food fussiness (being highly selective about foods [5, 6]), satiety responsiveness (terminating eating in response to fullness cues), slowness in eating (taking a long time to eat [5, 6]), picky eating (rejection of familiar and unfamiliar foods [24]), and food neophobia (fearing new or unfamiliar foods [25]). Individual food avoidance behaviors have been consistently associated with lower diet variety and quality among young children [24], and associated with lower child BMI for some behaviors but not for others. In particular, child BMI does not show consistent associations with food neophobia and picky eating (see Brown [26] for a review), but has been inversely associated with parent reports of satiety responsiveness [9–13, 15, 27] slowness in eating [10, 12, 13, 15] and food fussiness [10, 11, 13, 15, 26] in studies involving preschool-aged children.

Although the aforementioned range of behaviors are assumed to represent tendencies towards food approach and food avoidance [3, 4], scientific understanding of how individual eating behaviors aggregate within children to reflect multi-dimensional dispositions toward eating is limited. Research to date has largely utilized “variable-centered” approaches that evaluate individual eating behaviors separately, in isolation of one another, to identify relative contributions in predictive relationships with child dietary and weight outcomes [28, 29]. There has been relatively little research to understand whether and how individual eating behaviors aggregate within individuals to represent distinct typologies or profiles of eating behavior. Person-centered approaches can provide rich phenotypes of the “whole child” that are not possible to determine with variable-centered approaches. Characterizing fundamental differences in the way children approach eating may have utility for identifying children at risk for poor dietary quality (e.g., low fruit and vegetable intake, high intake of saturated fats and added sugars) and weight outcomes (i.e., underweight, overweight, obesity). Eating behavior profiles that can be identified by caregiver report may also have utility for tailoring interventions to address obesity [30] for children who exhibit biologically-influenced predispositions to overeat [31].

Few studies to date [32–39] have employed “person-centered” [40] analytical approaches to understanding eating behavior of the whole child. Of those that have, most have focused on older children [34, 36, 37] and/or focused narrowly on specific dimensions of eating (e.g., picky eating) [35, 39]. To our knowledge, only one study to date has focused exclusively on preschool-aged children [35]; that analysis focused on picky/fussy eating phenotypes among 4-year-old children in a large Danish birth cohort. The absence of empirical description of eating behavior profiles across a wide spectrum of behaviors during early childhood is a notable scientific gap given the importance of these years for the development of eating behaviors [41].

The main objective of this research was to empirically characterize multi-dimensional eating behavior profiles across a broad spectrum of individual eating behaviors in an ethnically and racially diverse sample of preschool-aged children with low-income backgrounds. This research represents the first comprehensive study of eating behavior profiles focused on preschool-aged children with low-income backgrounds for whom access to healthy foods may be limited relative to energy-dense, nutrient-poor foods [42, 43] and risks of poor diet quality [44, 45] and weight outcomes [46, 47] are elevated. A secondary objective was to obtain evidence of validity by evaluating associations of eating behavior profiles with child weight status and temperament. Temperament refers to basic predispositions around reactivity and self-regulation that, like appetite, are believed to reflect an interplay of genetic, biological, and environmental factors [48–50]. While there has been considerable recent interest in understanding the role of temperament in children’s self-regulation of appetite [49, 51–56], the extent to which eating behaviors are directly shaped by temperament is unclear [57]. Consistent with current theoretical perspectives, we hypothesized that empirically derived eating behavior profiles would broadly reflect dispositions towards food approach and food avoidance and would be aligned with weight outcomes [4] and differentiated by temperamental characteristics [57].

## Methods

### Design and Setting

This was a secondary, cross-sectional analysis of baseline data from preschool-aged children and their primary caregivers from the Growing Healthy [58] and ABC Preschool [59] studies. The Growing Healthy Study was a randomized controlled obesity prevention trial among preschoolers attending Michigan Head Start (USA) from 2011–2015 (clinicaltrials.gov identifier: NCT01398358). ABC Preschool was an observational cohort study investigating associations between stress and eating among preschool aged children from families with low incomes attending Michigan Head Start (USA) from 2009–2011. In each study, parents provided written informed consent for themselves and for their children.

### Participants

Parent–child dyads for each study were recruited through Head Start programs in Michigan. Head Start is a federally-funded preschool program for high-risk families in the US with low incomes. Participating children were 3–4 years of age at enrollment. Exclusion criteria for both studies included the child having a significant developmental disability, child being in foster care, or parent non-fluency in English. Additional exclusion criteria for the ABC Preschool study included parent with a 4-year college degree (as a proxy of higher socio-economic status), child having serious medical problems or history of food allergies, gestational age < 35 weeks, and significant perinatal or neonatal complications.

### Measures

To assess children’s eating behaviors, parents completed the widely-used Children’s Eating Behavior Questionnaire (CEBQ; 8 subscales, 34 items) [5]. Responses on a 5-point scale (1 = never to 5 = always) are averaged by subscale. Following the approach of Tharner et al. [35], the Emotional Overeating, Emotional Undereating, and Desire to Drink subscales were not included in the analysis. Thus, we focused on the following 5 subscales, with internal consistencies estimated from the current pooled sample: Food Responsiveness (5 items, *α* = 0.80), Enjoyment of Food (4 items, *α* = 0.85), Food Fussiness (6 items, *α* = 0.88), Slowness in Eating (4 items, *α* = 0.70), Satiety Responsiveness (5 items, *α* = 0.72). A recent systematic review of 27 studies revealed consistent cross-sectional associations of individual CEBQ subscales used in this analysis with BMI z-scores among children 1 to 13 years [60].

To assess temperament, parents completed items from the Children’s Behavior Questionnaire Short Form (CBQ; 15 subscales, 94 items) [61, 62]. Responses on a 7-point scale (1 = extremely untrue to 7 = extremely true) were averaged for each subscale. The present analysis utilized the following 3 subscales [61, 62] completed in both studies, with estimated internal consistencies from the current pooled sample: Anger-Frustration (i.e., amount of negative affect related to interruption of ongoing tasks or goal blocking; 6 items, *α* = 0.77), Impulsivity (i.e., lower speed of response initiation; 6 items, *α* = 0.53), and Inhibitory Control (i.e., the ability to actively inhibit or delay a dominant response to achieve a goal; 6 items, *α* = 0.68). The CBQ has shown strong inter-rater agreement between parents and strong stability over time [62], is predictive of behavior in the laboratory [63], and correlates with physiological measures of behavioral reactivity in young children (e.g., cortisol, vagal tone) [64].

Research staff measured children’s weight and height in duplicate without shoes or heavy clothing using a Detecto Portable Scale Model #DR550C and a Seca 213/217 portable stadiometer. Children’s body mass index BMI *z*-score and percentiles were derived using United States Centers for Disease Control reference growth curves by age and sex [65]. Children’s weight status was categorized as underweight (BMI < 5th percentile), normal weight (BMI ≥ 5^th^ and < 85th percentiles), overweight (BMI ≥ 85th and < 95th percentiles), and obese (BMI ≥ 95th percentile).

Socio-demographic characteristics known to be associated with dietary intake [66, 67] and/or weight [47]—sex, race and ethnicity, income, education, and food insecurity–were used to describe the sample and characterize profile membership. Parents reported parent and child race and ethnicity, child sex, child birth date (from which age was calculated), family structure, and annual household income. Income was divided by the federal poverty line for a family of a specific size to generate the income-to-needs ratio. An income-to-needs ratio < 1.00 indicates that the family was living below the federal poverty line. Food insecurity was categorized as food secure versus not food secure based on the US Department of Agriculture Food Security Scale [68].

### Statistical analysis

Descriptive statistics were generated for all variables of interest. This analysis included participants who had data on CEBQ variables (*n* = 1004); of these, 5 participants were missing 1 to 3 of the 5 contributing CEBQ subscales. To identify latent profiles of children’s eating behaviors, we conducted a latent profile analysis (Mplus 8.4) based on the mean CEBQ subscale scores for Enjoyment of Food, Food Responsiveness, Satiety Responsiveness, Slowness in Eating, and Food Fussiness. The optimal number of classes in LPA was derived based on the Bayesian Information Criteria (BIC) and the Lo-Mendell-Rubin test [69]. Posterior probabilities of classification were estimated to characterize the likelihood of correctly classifying individuals in profiles [70], with a recommended threshold of 80% [71]. Bivariate analyses, including Chi-square and analysis of variance, were used to compare mean BMI *z*-scores, weight status, temperament, and demographic characteristics across profiles. Significance values of *P* < 0.05 were considered to denote statistical significance.

## Results

Of 1004 participating parent–child dyads, 62% were from the Growing Healthy cohort, and 38% were from the ABC Preschool cohort. The combined analytic sample of children was racially and ethnically diverse (51.2% non-Hispanic White, 24.6% non-Hispanic Black, 11.7% Hispanic (any race)) and about half (50.7%) were female, with a prevalence of underweight, normal weight, overweight, and obesity of 2.2%, 62.0%, 18.7%, and 17.1%, respectively. Among participating parents, > 95% were mothers. Approximately half of parents reported education beyond high school (52.3%) and being married or partnered (56.5%). Mean household income-to-needs ratio was below the poverty line (0.86) and nearly one-third of parents (32.7%) reported household food insecurity. Table 1 presents mean CEBQ subscale scores for the sample.

**Table 1:** Children’s Eating Behavior Questionnaire (CEBQ) subscale means

	**Total Sample *n* = 1004**
CEBQ Subscale	**Mean (SD)**
Food Responsiveness	2.65 (.94)
Enjoyment of Food	3.91 (.80)
Satiety Responsiveness	2.91 (.70)
Slowness in Eating	2.97 (.77)
Food Fussiness	2.78 (.92)

Mean item score on a 5-point scale (1 = never to 5 = always)

**Fig. 1:**
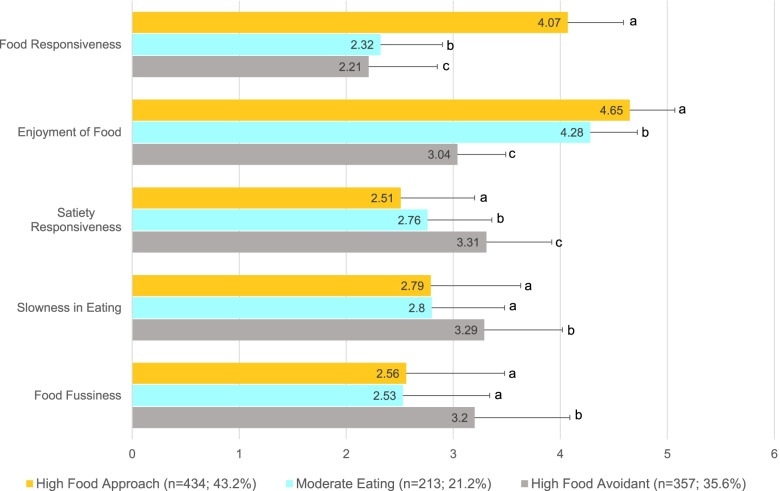
Children’s Eating Behavior Questionnaire (CEBQ) subscale scores by eating behavior profile. Legend. Mean item score (SD) on a 5-point scale (1 = never to 5 = always). For each CEBQ subscale, different superscripts denote mean differences between eating behavior profiles (all *P* < .05)

The optimal number of profiles was 3, BIC = 12,893.1 was lowest and Lo-Mendell-Rubin *P* = 0.0001 showed significant improvement comparing 2 vs. 3 classes but was non-significant *P* = 0.295 when comparing 3 vs. 4 classes. The posterior probabilities of classification were 84% for profiles 1 and 2 and 83% for profile 3. The three identified profiles reflected High Food Avoidance (Profile 1; 35.6%), High Food Approach (Profile 2; 21.2%), and Moderate Eating Behavior (Profile 3; 43.2%). As shown in the Fig. 1, children in the High Food Approach profile had higher levels of Food Responsiveness and Enjoyment of Food and significantly lower levels of Satiety Responsiveness compared to the other profiles (all *P* < 0.05). Alternatively, children in the High Food Avoidance profile showed higher levels of Satiety Responsiveness, Slowness in Eating, and Food Fussiness as well as significantly lower Food Responsiveness and Enjoyment of Food compared to the other profiles (all *P* < 0.05). Children in the Moderate Eating Profile had moderate or intermediary levels of Food Responsiveness, Enjoyment of Food, and Satiety Responsiveness (all *P* < 0.05) relative to the other profiles. Additionally, children in the Moderate Eating profile had levels of Slowness in Eating and Food Fussiness that were lower than children in High Food Avoidance profile (all *P* < 0.05) but did not differ from children in the High Food Approach profile.

Table 2 presents associations of eating behavior profiles with children’s temperament and weight status. Children in the High Food Avoidance profile had lower levels of Impulsivity and intermediary levels of Inhibitory Control relative to children in the other profiles (all *P* < 0.05). In contrast, children in the High Food Approach profile had lower Inhibitory Control than children in the other profiles (both *P* < 0.05) and lower Impulsivity than children in the High Food Avoidance profile but not the Moderate Eating profile. Finally, children in the Moderate Eating profile had higher Inhibitory Control and lower Anger/Frustration than children in the other profiles (all *P* < 0.05).

**Table 2:** Temperament and weight status by eating behavior profile

	**Total Sample**	**High Food Avoidant**	**High Food Approach**	**Moderate** **Eating**	***P***
		**Profile 1**	**Profile 2**	**Profile 3**	
	***N* = 1004**	***n* = 357 (35.6%)**	***n* = 213 (21.2%)**	***n* = 434 (43.2%)**	
Temperament					
CBQ Anger/Frustration, *M* (*SD*)	4.74 (1.21)	4.79 (1.24)^a^	4.92 (1.26)^a^	4.61 (1.15)^b^	.01
CBQ Impulsivity, *M* (*SD*)	4.76 (.88)	4.62 (.88)^a^	4.84 (.93)^b^	4.83 (.84)^b^	.001
CBQ Inhibitory Control, *M* (*SD*)	4.55 (1.02)	4.49 (.98)^a^	4.29 (1.09)^b^	4.74 (.98)^c^	< .0001
Weight					
BMI *z*-score, *M* (*SD*)	.67 (1.13)	.38 (1.14)^a^	.90 (1.20)^b^	.80 (1.03)^b^	< .0001
Weight status, *n* (%)					< .0001*
With Obesity	169 (17.1)	36 (1.3)	48 (23.2)	85 (19.7)	
With Overweight	185 (18.7)	56 (16.0)^a^	41 (19.8)^b^	88 (2.4)^a,b^	
With Underweight/Normal weight^†^	636 (64.2)	259 (73.8)^a^	118 (57.0)^b^	259 (6.0)^b^	

Children’s Behavior Questionnaire (CBQ)

Differing superscript letters indicate differences between eating behavior profiles (*P* < .05); superscript letters that are the same indicate no difference between eating behavior profiles

^*^Predicting profile membership from 3-category weight status, using multinomial regression and “with obesity” as the referent

^†^Underweight (2.2%) and normal weight (62.0%) were combined for analysis given the small cell size of underweight

Children in the Food Avoidance profile had lower BMI*z* than children in the High Food Approach and Moderate Eating profiles, respectively (all *P* < 0.05). Weight status also differed by profile membership (*P* < 0.001), with normal/underweight weight status being more common in the High Food Avoidance profile than High Food Approach and Moderate Eating profiles (73.8% vs. 57.0% and 60.0%, respectively), and obesity being more common in the High Food Approach profile than High Food Avoidance and Moderate Eating profiles (23.2% vs. 10.3% and 19.7%, respectively).

Table 3 presents demographic characteristics by eating behavior profiles. The profiles did not differ with regard to child age (*P* = 0.50), sex (*P* = 0.14), race and ethnicity (*P* = 0.13), parent race and ethnicity (*P* = 0.25) or education (*P* = 0.69), family income-to-needs ratio (*P* = 0.24) or family structure (*P* = 0.48). However, food insecurity differed by profile membership, such that household food insecurity was more common in the High Food Approach profile than the others (39.7% vs. 32.4% and 29.5%, High Food Approach vs. High Food Avoidance and Moderate Eating Behavior, respectively, all *P* < 0.05).

**Table 3:** Family demographic characteristics by eating behavior profile

	**Total Sample**	**High Food Avoidant**	**High Food Approach**	**Moderate** **Eating**	***P***
		**Profile 1**	**Profile 2**	**Profile 3**	
	***N* = 1004**	***n* = 357 (35.6%)**	***n* = 213 (21.2%)**	***n* = 434 (43.2%)**	
Child age, months, *M* (*SD*)	49.7 (6.3)	5.0 (6.3)	49.4 (6.7)	49.7 (6.1)	.50
Child sex, *n* (%)					.14
Male	494 (49.3)	174 (48.9)	94 (44.1)	226 (52.3)	
Female	507 (50.7)	182 (51.1)	119 (55.9)	206 (47.7)	
Child race and ethnicity, *n* (%)					.13
White, Non-Hispanic	511 (51.1)	183 (51.6)	113 (53.1)	215 (49.8)	
Black, Non-Hispanic	246 (24.6)	77 (21.7)	61 (28.6)	108 (25.0)	
Hispanic and/or other race*	243 (24.3)	95 (26.8)	39 (18.3)	109 (25.2)	
Maternal race and ethnicity, *n* (%)					.25
White, non-Hispanic	617 (61.6)	228 (64.0)	122 (57.6)	267 (61.5)	
Black, non-Hispanic	246 (24.6)	74 (2.8)	60 (28.3)	112 (25.8)	
Hispanic and/or other race*	139 (13.9)	54 (15.2)	30 (14.2)	55 (12.7)	
Maternal education, *n* (%)					.69
≤ HS Grad/GED	477 (47.8)	173 (48.9)	112 (52.6)	192 (44.4)	
> HS Grad/GED	522 (52.3)	181 (51.1)	101 (47.4)	240 (55.6)	
Marital status, *n* (%)					.48
Single parent	392 (43.6)	126 (40.7)	85 (44.3)	181 (45.5)	
Married	260 (28.9)	88 (28.4)	54 (28.1)	118 (29.7)	
Committed relationship	248 (27.6)	96 (31.0)	53 (27.6)	99 (24.9)	
Household income-to-needs ratio, *M* (*SD*)	.86 (.64)	.89 (.71)	.79 (.56)	.87 (.62)	.24
Household Food Insecurity, *n* (%)					.04^†^
Food Secure	665 (67.3)	240 (67.6)^a, b^	126 (60.3)^b^	299 (70.5)^a^	
Food Insecure	323 (32.7)	115 (32.4)	83 (39.7)	125 (29.5)	

Differing superscript letters indicate differences between eating behavior profiles (*P* < .05); superscript letters that are the same indicate no difference between eating behavior profiles post-hoc analyses

^*^Hispanic and non-Hispanic multiracial or other race were combined for analysis given the small sample sizes

^†^Predicting profile membership using multinomial regression and “food insecure” as the referent

## Discussion

The findings of this study provide the first empirical characterization of eating behavior profiles in a large, racially and ethnically diverse, sample of US preschool-aged children with low-income backgrounds. Based on parental reports of preschool-aged children’s eating behavior, just over one-third of children had profiles reflecting high food avoidance, whereas under a quarter of children had profiles reflecting high food approach. The remainder of children, representing the largest proportion of the sample (~ 40%), were characterized as having moderate levels of those behaviors. These findings extend previous work by providing new evidence that food avoidance and food approach represent empirically distinct, multi-dimensional eating profiles that are observable as early as preschool, can be identified based on parent-report, and are systematically related to temperament and weight status.

Eating behavior profiles were uniquely differentiated by the prominence of individual behaviors. Specifically, high food responsiveness was an especially robust feature of the High Food Approach profile, differentiating children in this group from the High Food Avoidance and Moderate Eating profiles especially strongly. Mean CEBQ Food Responsiveness scores for children in the High Food Approach profile indicated parental ratings of “often” in response to items such as “always asking for food”, “would eat too much, most of the time”, “always have food in their mouth if they could”, and “finds room to eat their favorite food even if they are full.” In contrast, mean CEBQ Food Responsiveness scores among children in the High Food Avoidance and Moderate Eating profiles indicated parental responses of “rarely” for the same items. The High Food Approach profile also uniquely contrasted with High Food Avoidance and Moderate Eating profiles in having greater mean CEBQ Enjoyment of Food and lower Satiety Responsiveness, though neither of these features differed as dramatically as Food Responsiveness. Alternatively, low enjoyment of food was an especially robust feature of the High Food Avoidance profile, that differentiated children in this group from the other profiles. Mean CEBQ Enjoyment of Food scores for children in the High Food Avoidance profile indicated parental ratings of “sometimes” in response to items such as “love food”, “interested in food”, “look forward to mealtime”, and “enjoy eating” whereas mean Enjoyment of Food scores for the other two profiles corresponded to ratings of “often.” Food avoidant children also contrasted with high food approach and moderate children in their lower CEBQ Food Responsiveness, higher Satiety Responsiveness, greater Slowness in Eating, and greater Food Fussiness, though none of these features differed as dramatically as Enjoyment of Food. That the High Food Approach and High Food Avoidance profiles were primarily distinguished by a single characteristic (e.g., high CEBQ Food Responsiveness in the case of food approach) suggests the potential value of identifying salient indicators of risk within profiles. However, whether single indicators can perform as well as multi-dimensional eating profiles in identifying children’s susceptibility to dietary, parenting, and obesity-related outcomes is an important empirical question that merits further inquiry.

In this study, the High Food Avoidance profile had lower BMI *z*-scores than the other profiles and the lowest percentage of children with obesity. These associations are generally consistent with previous studies of individual behaviors of which the profiles are comprised [4]. The association of food avoidance with lower weight is also consistent with findings of the only other study of eating behavior profiles among preschool-aged children [35]. In a Danish birth cohort study of 4914 4-year-old children that focused on picky/fussy eating, fussy eaters (5.6% of the sample) had lower BMI than non-fussy eaters [35]. Our findings also mirror those of a large birth cohort study of eating profiles across a broader period of childhood (from 15 months to 10 years of age) [38]. In that study of 12,048 UK children, 16 profiles were identified, with an “early and increasing overeating” class associated with higher BMI*z* at age 11 and “persistent undereating” and “persistent fussiness” classes associated with lower BMI*z*. Finally, a previous longitudinal analysis of picky eating profiles among children 4 to 9 years of age from the same cohort used in this analysis, showed lower BMI*z* among children with medium and high trajectories of picky eating [39]. The present research provides new evidence that distinct multi-dimensional typologies reflecting food avoidance as well as food approach can be differentiated by weight status with as few as 3 profiles. That the largest proportion of children were classified by a moderate eating profile also suggests that the typical child will have tendencies towards avoidance and approach that are less pronounced than children with high avoidance or approach. It is important to note that the data-driven nature of profile analysis, as well as the number and nature of variables used to identify profiles, inherently constrains generalizability; the number of eating behavior profiles identified in previous studies of older children has ranged from 3 to 16 [32–38]. Additional research is needed to determine whether subpopulations exist within food avoidant and food approach profiles that are clinically important to differentiate.

Wardle and colleagues [6, 14, 72, 73] formulated the behavioral susceptibility theory of obesity which holds that appetitive traits have a strong genetic component [72] and confer susceptibility to obesity by guiding children’s behavior in a given eating environment to promote excessive energy intake. The existence of eating behavior profiles extends the concept of behavioral susceptibility by suggesting that such traits aggregate to shape dispositions toward eating that confer susceptibility to obesity. Much of the work on behavioral susceptibility to date has focused on evaluating the association of appetitive traits with weight status [74]. Relatively less is known about how young children’s behavioral predispositions toward eating influence the way children interact with social and physical environments surrounding eating, including food security and food parenting practices, to shape dietary and weight outcomes. In this study, household food-insecurity was more prevalent among children with high food approach profiles. This preliminary finding suggests that food-insecure environments may intensify food motivated behavior in children. For instance, lower neighborhood income-levels have been associated with a higher density of “unhealthy” food outlets (e.g., convenience stores) [43]. Additionally, greater household food insecurity among adults has been associated with greater intake of ultra-processed foods [75]. The developmental sciences have long-recognized that optimal child outcomes are most likely to occur when caregiving is matched to the child’s temperament and environmental conditions in which development occurs (i.e., “goodness of fit” [76]). Research is needed to better understand how children’s fundamental dispositions towards eating can inform the types of approaches that best support healthy eating behaviors and growth in diverse socioeconomic and physical environments.

To our knowledge, this is also the first study to evaluate the association of eating behavior profiles with temperament. Russell and Russell proposed a biopsychosocial model of pathways to overweight and obesity in childhood [74], in which temperament shapes dietary and weight outcomes via influences on children’s eating behavior as well as indirectly through shaping parental cognitions and behaviors towards the child. For instance, children with greater temperamental negative affectivity may elicit greater use of food to manage negative affect, which in turn might shape emotion-based eating behavior in the child. In this study, children with high food avoidance profiles had lower impulsivity, whereas children with high food approach profiles had low inhibitory control compared to the other profiles. These findings are generally consistent with a number of recent studies that have evaluated associations of temperament with individual food avoidance and approach behaviors among children [49, 51–56]. For instance, in a longitudinal study of 997 Norwegian children followed from ages 4 to 10 years, higher negative affectivity (of which anger/frustration is a component) was associated with higher food approach and food avoidance behaviors, whereas greater surgency (of which impulsivity is a component) was associated with higher food approach behaviors, but lower food avoidance behaviors [54]. We also have previously reported similar cross-sectional positive associations of surgency among preschool-aged children with higher food responsiveness and enjoyment of food [77]. Collectively, these findings point to alignment of the dimensions of surgency with food approach profiles and dimensions of negative affectivity with food avoidance. Longitudinal evidence is needed to better understand the extent to which eating behavior profiles are shaped by temperament and may moderate associations with diet and weight related outcomes [78], including obesity. A somewhat separate but related issue for future research involves the extent to which parental perceptions of temperament and eating behavior are conflated in the approach to feeding and/or beliefs about the mutability of eating behaviors.

This analysis has both strengths and limitations that merit consideration. First, the strengths of this work include a relatively large sample size in an English-speaking, racially and ethnically diverse sample of young children with low-income backgrounds. Whether results generalize to Spanish-speaking parents, however, is unclear. Similarly, the focus of this research on children from low-income backgrounds has significance in efforts to promote health equity in underserved populations. In the present study, the prevalence of household food insecurity was highest among children with high food approach profiles. A previous qualitative study of family members of children from low-income households described food hiding, binge eating, and nighttime eating behaviors among food-insecure children with obesity [79]. Another recent analysis of eating behaviors and appetite among UK children at 16 months and 5 years of age showed prospective associations of low socioeconomic status with food responsiveness and emotional overeating [80]. These findings collectively raise interesting questions for future research about the impact of low levels of household income and food security on food motivated behavior among children. However, findings may not generalize to children from higher-income backgrounds. Reliance on parental-report measures of children’s eating behavior is another limitation of this research. Future research should incorporate objectively measured eating behaviors to reduce potential method bias as well as to provide richer or deeper phenotypes of children’s behavior. Finally, analyses of child temperament were limited to select dimensions that were common to both studies. Future studies should consider a broader range of constructs [81]. Further, the low internal consistency of the Impulsivity subscale is a limitation that has been noted previously in low-income and racial/ethnic minority children [61].

## Conclusions

In conclusion, these findings suggest that empirically distinct, multi-dimensional eating behavior profiles reflecting food avoidance and approach are associated with weight and temperament and emerge early in childhood. These findings highlight the potential utility of “whole person” approaches and underscore fundamental differences in the way children approach eating. Utilizing person-centered approaches may be useful in moving towards parsimonious definitions of eating related phenomena that involve multiple facets, such as those described in various definitions of picky eating [82]. Identifying eating typologies that can be reliably observed by parents and practitioners may also have future utility for tailoring health promotion and obesity prevention efforts to the needs of different types of eaters. To this end, additional research is needed to 1) determine the best methods for identifying multi-dimensional eating behavior profiles among children and 2) to evaluate whether the effectiveness of health promotion and obesity prevention efforts is improved by taking into account children’s unique dispositions toward eating.

## Data Availability

The datasets used and/or analyzed during the current study are available from the corresponding author on reasonable request.

## Abbreviations

BIC: Bayesian information criterion
BMI: Body mass index
CBQ: Children’s Behavior Questionnaire
CEBQ: Children’s Eating Behavior Questionnaire
