# Correction: Characteristics of eating behavior profiles among preschoolers with low-income backgrounds: a person-centered analysis

**DOI:** 10.1186/s12966-022-01341-w

**Published:** 2022-08-08

**Authors:** Jennifer Orlet Fisher, Sheryl O. Hughes, Alison L. Miller, Mildred A. Horodynski, Holly E. Brophy-Herb, Dawn A. Contreras, Niko Kaciroti, Karen E. Peterson, Katherine L. Rosenblum, Danielle Appugliese, Julie C. Lumeng

**Affiliations:** 1 Center for Obesity Research and Education, College of Public Health, Temple University, Philadelphia, PA USA; 2 USDA/ARS Children’s Nutrition Research Center, Baylor College of Medicine, Houston, TX USA; 3 Department of Health Behavior and Health Education, School of Public Health, University of Michigan, Ann Arbor, MI USA; 4 College of Nursing, Michigan State University, East Lansing, MI USA; 5 Department of Human Development and Family Studies, Michigan State University, East Lansing, MI USA; 6 Health and Nutrition Institute, Michigan State University Extension, East Lansing, MI USA; 7 Department of Pediatrics, University of Michigan Medical School, Ann Arbor, MI USA; 8 Department of Nutritional Sciences, School of Public Health, University of Michigan, Ann Arbor, MI USA; 9 Department of Psychiatry, University of Michigan Medical School, Ann Arbor, MI USA; 10 Appugliese Professional Advisors, LLC, North Easton, MA USA

**Correction: Int J Behav Nutr Phys Act 19, 91 2022**

10.1186/s12966-022-01323-y

Following publication of the original article [1], the authors identified an error in Fig. 1. The correct figure is given below.

**Fig. 1:**
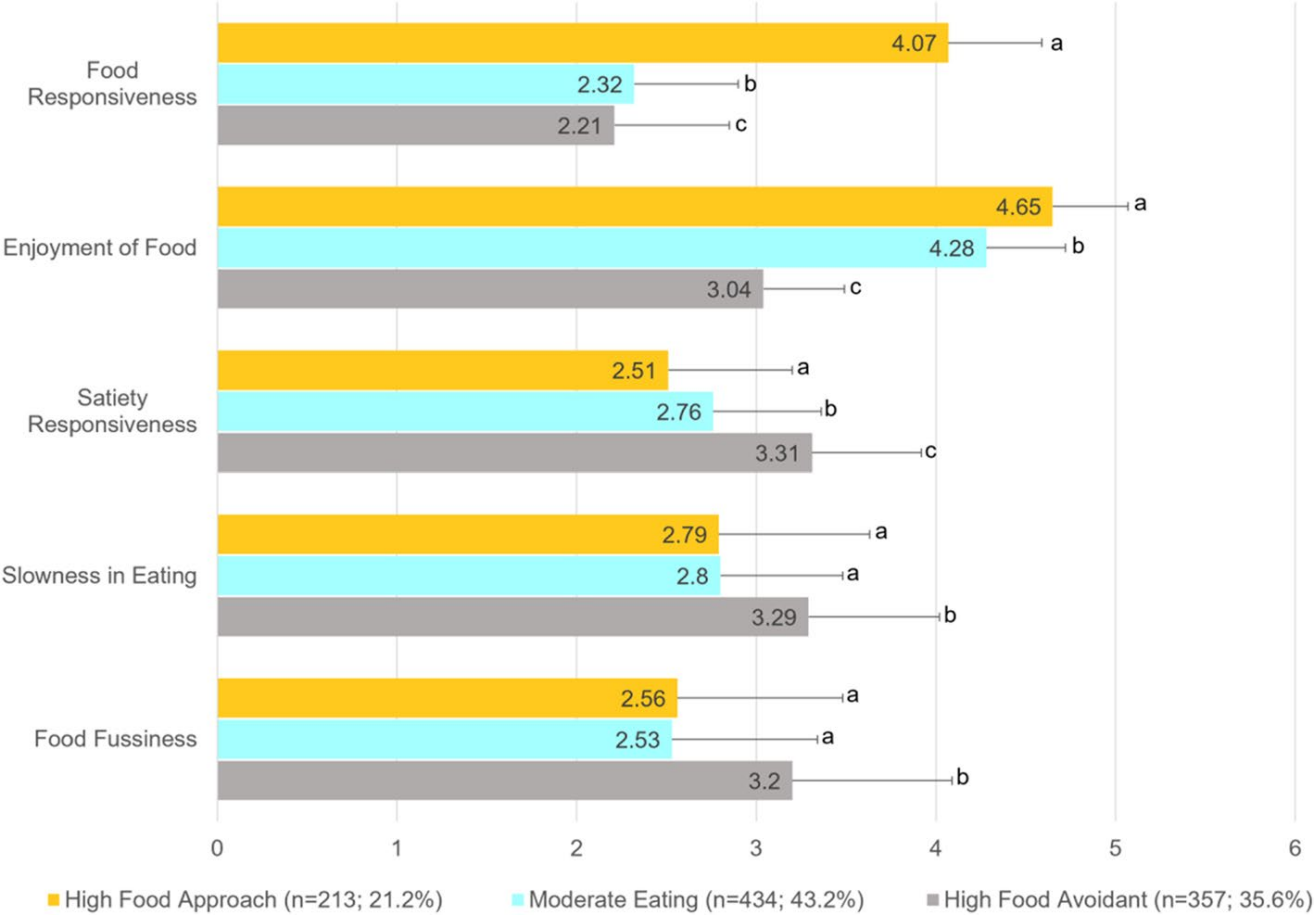
Children’s Eating Behavior Questionnaire (CEBQ) subscale scores by eating behavior profile. Legend. Mean item score (SD) on a 5-point scale (1 = never to 5 = always). For each CEBQ subscale, different superscripts denote mean differences between eating behavior profiles (all *P* < .05)

The original article [1] has been corrected.
